# The Next Paradigm Shift in the Management of Clear Cell Renal Cancer: Radiogenomics—Definition, Current Advances, and Future Directions

**DOI:** 10.3390/cancers14030793

**Published:** 2022-02-04

**Authors:** Nikhil Gopal, Pouria Yazdian Anari, Evrim Turkbey, Elizabeth C. Jones, Ashkan A. Malayeri

**Affiliations:** 1 Urologic Oncology Branch, National Cancer Institute, National Institutes of Health, 10 Center Drive, Bethesda, MD 20814, USA; 2 Radiology and Imaging Sciences, Clinical Center, National Institutes of Health, 10 Center Drive, Bethesda, MD 20814, USA; pouria.yazdian@nih.gov (P.Y.A.); evrim.turkbey@nih.gov (E.T.); ejones@cc.nih.gov (E.C.J.); ashkan.malayeri@nih.gov (A.A.M.)

**Keywords:** clear cell kidney cancer, radiogenomics, radiomics, machine learning, gene expression

## Abstract

**Simple Summary:**

Radiogenomics is the science of studying imaging–pathology associations on a genomic level. With the potential for improved non-invasive characterization of tumors to predict survival; metastasis; and/or treatment response, it is important for clinicians to have a basic appreciation of this nascent field. The genetic basis for clear cell kidney cancer is more well-defined than many other malignancies, making it an ideal target for radiogenomic analysis. We first define the field of radiogenomics in diagnostic radiology, demonstrating that image biomarkers can be derived either qualitatively or quantitatively, the latter of which often employs machine learning. We then summarize existing literature establishing relationships between image features and single or multiple gene expression patterns in clear cell renal cell carcinoma. Finally, we outline limitations of the scope and methodology of current radiogenomic studies in ccRCC and propose future directions for this field to progress from an experimental setting into the mainstream clinical workflow.

**Abstract:**

With improved molecular characterization of clear cell renal cancer and advances in texture analysis as well as machine learning, diagnostic radiology is primed to enter personalized medicine with radiogenomics: the identification of relationships between tumor image features and underlying genomic expression. By developing surrogate image biomarkers, clinicians can augment their ability to non-invasively characterize a tumor and predict clinically relevant outcomes (i.e., overall survival; metastasis-free survival; or complete/partial response to treatment). It is thus important for clinicians to have a basic understanding of this nascent field, which can be difficult due to the technical complexity of many of the studies. We conducted a review of the existing literature for radiogenomics in clear cell kidney cancer, including original full-text articles until September 2021. We provide a basic description of radiogenomics in diagnostic radiology; summarize existing literature on relationships between image features and gene expression patterns, either computationally or by radiologists; and propose future directions to facilitate integration of this field into the clinical setting.

## 1. Introduction

Beginning in the late 1980s, our understanding of the pathology of kidney cancer has gradually evolved beyond characterization of histological patterns to identification of specific genetic changes [1,2]. Discovery of pathologically relevant genetic pathways has allowed for discrimination both between and among renal cancer subtypes. The ultimate goal of these endeavors is to create a more personalized approach to predicting disease prognosis and response to treatment. With improved ability to characterize image features, particularly through advances in machine learning, diagnostic radiology is also primed to enter personalized medicine through the field of radiogenomics. Here, we define this nascent field and review available studies in clear cell kidney cancer involving the association of single-gene mutations as well as more complex gene expression patterns with imaging phenotypes.

## 2. What Is Radiogenomics?

Radiogenomics is the science of identifying the associations between imaging features of a lesion and the underlying genomic signatures. For instance, by developing radiogenomics signatures, one can predict the tumor response to treatment by combining the imaging findings and genomic data. This process can also be used to decode the genetic makeup of a mass seen on imaging that fits the radiogenomic profile developed for that specific mass subtype [3,4,5]. One of the advantages of this approach is a complete evaluation of the makeup of the mass as opposed to tissue sampling that only evaluates a small portion of the tumor, which may underestimate the dominant molecular pattern given intra-tumoral heterogeneity [6]. Thus, by identifying surrogate imaging biomarkers that represent distinct genotypes with prognostic significance, radiogenomics can improve traditional tumor genetic testing through more comprehensive tumor characterization via wider anatomic coverage. As with current biomarkers, these imaging phenotypes should have prognostic significance; that is, to better define, beyond size and growth rate criteria alone, appropriate candidates for active surveillance and/or systemic treatment regimens in the case of advanced disease.

**Figure 1:**
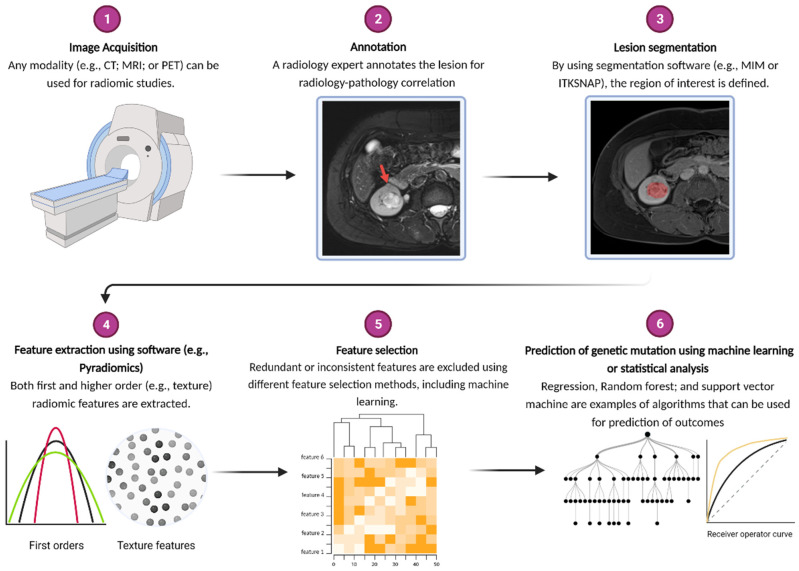
Outline of workflow for radiomic studies. Annotation is particularly important for multifocal masses to ensure matching of radiologically identified lesion with appropriate pathological specimen. Classification of machine learning algorithms is typically binary and thus analyzed using receiver operated curve (ROC), with area under the curve (AUC) used as benchmark for machine performance. Image created using BioRender.com (accessed on 26 November 2021).

Imaging characteristics can be obtained either qualitatively (i.e., discrete variables scored by one or more radiologists) or quantitatively. Some of the quantitative variables such as size and degree of contrast uptake/washout can be calculated by the clinicians, while more complex relationships between individual image pixels cannot be ascertained by the naked eye. Conversion of these relationships into mineable quantitative features is the practice of radiomics [5,7,8]. The region of interest (either a single slice or the full volume of the tumor) is marked within an image (segmentation) to be recognized by computer software for image feature extraction. Differential pixel intensities of an image can be captured into either first order features (i.e., frequency distribution of pixel intensities without any spatial information such as skewness or kurtosis) or higher order features (i.e., spatial relationship between different pixel intensities such as gray level discrimination matrix). Given the number of extracted features (at times exceeding 1000) and the assumed nonlinear relationship between features and the dependent variable (i.e., presence or absence of a genetic mutation), machine learning is often employed to establish such relationships. More specifically, the data are split into training and testing sets, with an assigned algorithm developing relationships among relevant features using training data. The ability of the model to accurately classify patients into discrete categories (i.e., mutation or no mutation) is employed on the test data, using the known mutation status as the comparator of efficacy. Typically, prior to model training, the number of extracted features is reduced, either by eliminating redundant features (i.e., those with high intra-class correlation) and inconsistent features (i.e., those not seen if tumor is segmented by a different radiologist), with or without the aid of machine learning. In summary, the steps of a radiomics algorithm are segmentation; feature extraction; feature selection; and, in most cases, machine learning. This workflow is summarized in Figure 1.

Compared to other malignancies, the genetic basis of clear cell kidney cancer is well-established, with a relative paucity of genes implicated in pathogenesis. Thus, kidney cancer is a prime target for initial application of radiogenomics. Below, we review available studies in clear cell renal cell cancer (ccRCC) radiogenomics, focusing on exploratory investigations into relationships between imaging features and mutations in single genes; gene expression patterns; methylation changes in specific genes; and microRNA expression. The goal of each of these investigations is to better predict relevant clinical endpoints, such as overall survival; development of metastasis; and treatment response.

**Figure 2:**
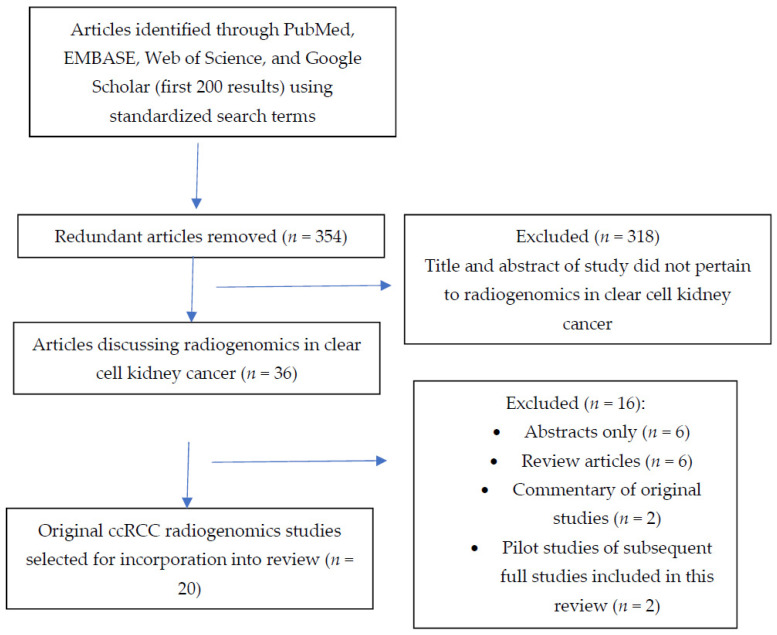
Flowchart demonstrating the search strategy and selection criteria for the articles included in this review.

We conducted the review using PubMed, EMBASE, Google Scholar, and Web of Science. We searched by title/abstract in the following databases using the search parameters: “artificial intelligence or radiomics or machine learning or deep learning or radiogenomics” AND “clear cell” AND “kidney or renal”. Articles published up to September 2021 were included. Eliminating redundant articles, 354 articles were identified from our search parameters. Titles from articles were screened out if they did not involve a correlation of imaging features to gene expression patterns. Through this manner, we identified 20 full text, original study articles that were incorporated into this review. See Figure 2 for a summary of the workflow for inclusion of studies for this review. Table 1 summarizes these studies with their relevant findings.

## 3. Associations between Image Features and Mutations in Single Genes Commonly Implicated in ccRCC

While mutations in Von-Hippel Lindau (VHL) gene have long been implicated in the pathogenesis of ccRCC [1,2], the Cancer Genome Atlas (TCGA) helped identify additional causative genes, including those in the chromosome 3p region adjacent to VHL, such as polybromo-1 (PBRM1); BRCA associated protein 1 (BAP1); and SET domain containing 2 (SETD2) [28]. Indeed, while 90% of sporadic clear cell kidney cancers are associated with 3p chromosomal deletions, a minority of these tumors have wild type VHL expression, indicating the independent role of other genes within this region in tumorigenesis. Additional relevant genes for ccRCC identified by TCGA include lysine specific demethylase 5C (KDM5C) and mucin 4 (MUC-4) [29,30]. Although the presence of a VHL mutation itself has not been shown to have any predictive or prognostic value, important clinical differences emerge with respect to the mutational status of other genes. For instance, PBRM1 mutational status may determine response to immune checkpoint therapy [31,32]; BAP1 mutations are associated with more aggressive tumors [28,33]; tumors with SETD2 and KDM5C mutations are linked to unfavorable prognosis in the localized setting [34,35,36]; and tumors with MUC4 mutation have a favorable prognosis [37].

Karlo and others [9] sought to assess whether mutations in VHL; KDM5C; SETD2; and/or BAP1 were associated with any image features from computed tomography (CT). A total of 233 patients from two cohorts (i.e., MSKCC and the Cancer Imaging Archive (TCIA)) with available CT and genomic analysis had their corresponding tumors scored on eight qualitative (e.g., presence of necrosis) and two quantitative (e.g., tumor size) features via consensus from three radiologists. Significant image-genotype correlations were seen with VHL, KDM5C, and BAP1 mutations. Tumors with VHL mutations were associated with a well-defined tumor margin; nodular enhancement; and presence of intratumoral vascularity. KDM5C and BAP1 mutations were more predominant in tumors with renal vein invasion. Finally, KDM5C mutant tumors tended to be hypo-enhancing relative to the renal cortex in the CT nephrographic phase.

Shinagare et al. [10] performed a similar type of hypothesis-generating study; here, 103 patients exclusively from the Cancer Imaging Archive (TCIA) had six imaging features on either contrast-enhanced CT (79% of cohort) or MRI assessed by three radiologists. For each feature, the median or most common score (depending on whether the variable was qualitative or quantitative) was used to determine an association with tumor genotype. Despite the overlap in image features and patients with Karlo et al. [9], different results were obtained. With respect to VHL; KDM5C; and BAP1 mutational status, there was a significant association only with BAP1. Namely, tumors with BAP1 mutations were more likely to have ill-defined margins and calcifications. Additionally, MUC-4 mutation was associated with an exophytic tumor growth pattern.

Despite the inconsistency in results between these two studies, plausible biological explanations can be ascertained for these surrogate imaging biomarkers. For instance, BAP1 mutations confer aggressive traits to renal tumors, which may increase the likelihood of renal vein invasion as well as promote de-differentiation and increased proliferation, both of which can account for a poorly visualized tumor margin. The unregulated HIF expression with VHL mutation, resulting in upregulation of angiogenesis factors, can explain the prominence of intratumoral vascularity seen in these tumors.

Greco et al. [11] sought to characterize differences, if any, between patients with VHL and KDM5C mutant tumors in terms of abdominal fat content. With 52 VHL and 10 KDM5C mutant tumors derived from the TCIA cohort, patients with KDM5C mutations had higher total and visceral abdominal fat content than those with VHL tumor mutations. The authors also included a cohort of patients with no renal tumors (*n* = 35) and noted that ccRCC overall is associated with higher total and visceral fat content. There is evidence that fat deposits in obese individuals may promote oncogenesis and tumor progression through a chronic inflammatory state created through adipokines [38,39], which may explain the study results, given the negative prognostic biomarker of localized KDM5C mutant tumors.

Apart from qualitative and quantitative scoring derived from radiologists, associations between image features and single gene alterations have also been studied using radiomics and machine learning. For instance, Feng et al. [12] used a random forest classifier to assign tumors from 54 TCIA patients (45 BAP1 wildtype and 9 BAP1 mutants) to either presence or absence of BAP1 mutation based on 58 quantitatively derived radiomics features, with an AUC of 0.77. Image features from this study were derived from the nephrogenic CT phase, with the most predictive being a higher order feature (gray level run length matrix—number of consecutive voxels of a similar gray level intensity within a given direction [8]. Kock et al. [13] also used a random forest classifier to predict BAP1 tumor mutational status but used an unenhanced CT for easier availability and improved homogeneity between image studies, the latter of which is relevant in the multi-institutional collaboration of TCIA. Utilizing CTs of 65 patients (13 BAP1 mutant tumors and 52 BAP1 wildtype tumors), the random forest classifier was trained on 6 selected features, achieving an AUC of 0.897. Although Ghosh et al. had previously shown features extracted from nephrographic phase as opposed to unenhanced phase to be most predictive of BAP1 mutation [14], it should be noted that different extracted features from Feng et al. [12] were used to train this model; indeed, the dominant feature class was first-order. Nevertheless, half of the selected features [13] were higher order, indicating that region of interest (ROI) analysis without taking into account the spatial relationship of encapsulated voxels (i.e., utilizing only first order features) was insufficient for optimal prediction of BAP1 mutation status.

In addition, to study results potentially being affected by the image phase used and features selected, the type of machine learning algorithm can have an impact on the predictive performance of the model classifier. For instance, Kocak et al. [15] assessed the differential performance of two algorithms (random forest classifier and artificial neural network) in predicting the presence or absence of a PBRM1 mutation. In studying 45 patients (29 PBRM1 tumor wild-type and 16 PBRM1 mutants) from the TCIA using the corticomedullary phase of CT, the random forest classifier outperformed the artificial neural network in predicting tumor genotype, with AUC of 0.987 and 0.925, respectively. In this study, a machine learning algorithm was used to select the extracted radiomic features as well as train the model using the selected features. In other words, while 828 initial features were extracted from the CT, the final features used to train the model classifier differed depending on the algorithm (i.e., 10 features selected by artificial neural network and 4 features by random forest classifier). Indeed, only three selected features were shared by both algorithms, accounting for discrepancy in results beyond the intrinsic properties of the algorithms themselves. Regardless, two out of the top three features most predictive of PBRM1 mutation status were a higher order for both types of model classifiers. Across both types of algorithms, tumors with the PBRM1 mutation had greater pixel heterogeneity of gray level intensity.

Rather than comparing different machine learning algorithms, Chen et al. [16] used six different types of classifiers to generate the composite probability of different tumor genetic mutations. Here, 43 selected features from corticomedullary phase CT scan (a total of 57 patients from TCIA) were used to train and test each model classifier (support vector machine; logistic regression; discriminant analysis; decision tree; K-nearest neighbor; and naïve Bayesian). The predictive capability of the multi-classifier algorithm was superior to any single classifier, with AUC for predicting VHL; PBRM1; and BAP1 mutations being 0.88; 0.86; and 0.93, respectively. The selected features common to all six classifiers that discriminated VHL mutational status were both first order (mean and kurtosis). Tumors with VHL mutation had lower mean voxel intensity and had less variation in pixel intensity values (i.e., less tailedness or kurtosis). On the other hand, a relatively equivalent proportion of first and higher order features were selected across all six classifiers for distinguishing PBRM1 mutation class. Finally, more higher order features were common to all six classifiers for BAP1 classification, with BAP1 mutant tumors having greater heterogeneity in terms of voxel intensity.

## 4. Beyond Mutations in Common Pathogenic Single Genes in Clear Cell Kidney Cancer: Establishing Image Biomarkers for Epigenetic, Regulatory, and Multiple Gene Expression Signatures

Despite single gene mutations being implicated in renal cancer pathogenesis, kidney cancer development is reliant not just on any one aberrant gene product, but also on changes in regulatory molecules for both the gene product and its downstream effectors. While our understanding of these modulators of gene expression is in its infancy, preliminary investigations into relationships between imaging features and these molecules have been conducted.

For instance, Marigliano et al. [17] sought to determine whether there was any association between intensity-based pixel features (e.g., mean pixel attenuation) of ccRCCs seen on contrast CT and the amount of mi-21-5p, a micro-RNA whose expression was previously shown to be correlated with poor cancer specific survival following RCC resection [40]. Unlike previous studies, image features were extracted from both the tumor and the surrounding normal renal parenchyma. In 20 patients, the authors found a significant positive correlation between change in miR-21-5p expression from tumor to adjacent normal parenchyma and degree of image entropy (i.e., variation in pixel intensity within the tumor) [17].

Another regulatory factor implicated in several carcinomas is RUNX3 (runt related transcription factor 3), which belongs to a family of transcription factors that modulate major developmental pathways [41,42]. Methylation of this tumor suppressor RUNX3 has been negatively associated with overall survival in other carcinomas [43,44]. Cen et al. [18] scored 106 ccRCCs from the TCIA cohort on 9 qualitative CT imaging features and found, on multivariate regression, that ill-defined tumor margin, left sided tumors, and presence of intratumoral vascularity significantly predicted elevated RUNX3 methylation levels (AUC of 0.725). Furthermore, patients with higher methylation levels had lower median overall survival. The laterality bias is difficult to explain, with additional validation needed, but intratumoral vascularity and ill-defined margin are both imaging markers associated with aggressive tumors, which is in line with the negative prognosis associated with RUNX3 methylation.

Other tumor suppressor genes that can be susceptible to methylation-induced silencing in RCC have been identified, such as Dickkopf1 (DKK1); WNT pathway regulatory genes; and secreted frizzed related protein (SFRP1) [45,46]. Through the TCGA, three DNA methylation subgroups in ccRCC (M1-M3) with prognostic implications were identified, with the M1 subtype found to have the worst overall survival [28]. In assessing tumors from 212 patients (180 ccRCC cases) from the TCIA cohort on 12 different qualitative CT imaging features, Yu et al. [19] noted that, on multivariate analysis, a long axis >7 cm and presence of necrosis was associated with the unfavorable M1 subtype, with an AUC of 0.68. While M2 subtype was mostly characterized by absence of necrosis, the presence of necrosis was a significant independent predictor of the M3 subtype on multivariate logistic regression, limiting the utility of that imaging parameter.

As illustrated above, characterizing tumors by a panel of molecular markers, as opposed to a single entity, may more accurately capture the full extent of their biological behavior. In this manner, Zhao et al. [47] described 259 genes that predicted survival after ccRCC surgery independent of grade; stage; and performance status, creating the so-called SPC (supervised principal components) gene signature. Jamshidi et al. [20] used available CT and genetic data from 70 patients from a single institution to develop a radiogenomic risk score (RRS) using the top 4 qualitative CT imaging features that were best associated with expression of genes within the SPC signature. This score was independently validated in 77 patients from the same institution at a later time point. In a separate phase II trial assessing the role of neoadjuvant bevacizumab prior to cytoreductive nephrectomy, RRS using pre-treatment CT features was able to predict radiological progression free survival after anti-angiogenic administration [4].

The Cancer Genome Atlas also helped identify four unique mRNA-based subgroups in clear cell renal cell cancer: m1–m4 [48]. For instance, M1 contains gene sets involved with chromatin remodeling and a higher proportion of PBRM1 mutations. On the other hand, higher deletions of PTEN are seen in the m3 subtype. Bowen et al. scored tumors from 177 patients from TCIA on 8 CT imaging features and noted that a well-defined tumor margin was a significant positive predictor of m1 subtype vs. others, whereas the opposite was true of the m3 subtype [21]. As seen in other qualitative studies, the margin status of the m1 subtype is in line with its prognostically favorable outcome with respect to overall survival.

Further genetic expression analysis of ccRCC tumors have revealed two distinct molecular subtypes that are captured by a 34-signature gene model (ClearCode34): ccA and ccB. CCA is characterized by upregulation of genes involved in angiogenesis, while ccB tumors have higher cellular differentiation activity (i.e., epithelial to mesenchymal signaling). CCB tumors are more aggressive, based on higher Furhman grade; increased nodal metastasis; and worsened cancer specific as well as overall survival [49,50]. Unfortunately, the utility of this biomarker is hindered due to high intra-tumoral heterogeneity [51], limiting radiogenomic studies derived from biopsy samples. Yin et al. [22] circumvented this problem by performing radiomic and genetic expression analysis on different areas of the tumor from the same patient. A total of 168 features were extracted from 23 tumor ROIs on a PET/MRI from 8 patients; using sparse partial least analysis (SPLA), 4 radiomic features (2 first order and 2 higher order) were selected and found to correctly classify the ccRCC molecular subtype 86.96% of the time.

Thus far, radiomic signatures have been linked to molecular factors with established prognostic associations; for instance, BAP1 mutation with aggressive tumor phenotype or ccB with worsened cancer specific survival. However, radiomic analysis can be used for gene discovery, with associated prognostic and therapeutic implications. That is, machine learning algorithms can group image features into those that are found to differ based on clinical outcomes such as metastasis free or overall survival. The genotype of tumors within each imaging group can then be interrogated to determine the underlying biology of different image classes, with identification of distinct genetic pathways helping to usher, for instance, development of new drugs.

Lee et al. [23] used three different machine learning algorithms (i.e., random forest classifier; logistic regression; and support vector machine) and a training set of 58 patients with a contrast CT prior to partial or radical nephrectomy to determine differential contributions of 4 selected image features (only 1 of which was higher order) towards prediction of post-surgical metastasis. This model was independently validated on 28 patients from the TCIA with an AUC of 0.89–0.95. Genetic expression analysis was performed on tumors, with specific image features correlating with genes involved with translation regulation; ECM interaction; focal adhesion; PI3K-AKT pathway; signaling by notch receptor 1 (NOTCH1); Wnt signaling pathway; and regulation of actin cytoskeleton. Differences in fibroblast growth factor expression and amount of T cells were found to correlate with image features, which have therapeutic implications (i.e., preferential FGFR inhibitor or immunotherapy for metastatic disease).

In a similar study, Zhao et al. [24] used nine radiomic features selected by machine learning (eight of which were higher order) to predict development of postoperative metastasis with AUC of 0.86. With genetic expression analysis and correlation with 9 image features, 19 gene signatures (ECM interaction; focal adhesion; and PI3K-AKT pathway were similar sets of genes from the previous study) were constructed that independently accurately predicted metastasis (AUC of 0.84). Additionally, Lin et al. [25] developed three distinct radiomic feature classes that, independent of tumor grade and patient age, differed based on overall survival from unenhanced CT scans of 160 patients. Genetic analysis revealed that classes differed based on underlying genetic mutations. For instance, class 1 with the lowest overall survival had reduced VHL mutation expression relative to the other two classes. Class 3 had higher FBN2 expression, which has been previously associated with improved overall survival [52,53]. Finally, Huang et al. [26] unearthed a gene expression module (comprised of 256 genes) that was associated with four selected radiomic features (75% higher-order) derived from 205 ccRCC patients from the TCIA. These genes mediate tumor angiogenesis, cell adhesion, and extracellular structure organization. The top four correlated genes within this module (RPS6KA2, CYYR1, KDR, and GIMAP6) were selected for incorporation into a machine learning algorithm. A decision classifier integrating both radiomic and genomic factors was a better predictor of 1-, 3-, and 5-year overall survival than a classifier using only radiomic features (5-year survival AUC 0.75 and 0.69, respectively).

## 5. Limitations and Future Directions

While radiogenomics has the potential to revolutionize a clinician’s diagnostic capabilities, several existing limitations in this field will need to be addressed to allow these advances to proceed beyond the experimental setting. First, many of the institutional-based studies fail to have an external validation set from an outside institution, limiting the generalizability of their findings. In a recent review, only 7% of studies utilizing radiomic analysis of renal masses had this type of validation [54].

Despite not having an independent validation set, studies attempt to nonetheless seek generalizability by relying on cohorts from TCIA, which are comprised of images from multiple institutions. However, as institutions differ in image processing protocols, a different problem emerges, particularly for radiomic analysis, with the type and quantity of features extracted dependent on the specific way an image is acquired and processed (e.g., number of slices used for segmentation) [5,54].

A significant time burden in the radiomics workflow is manual segmentation, especially if more than one slice is considered. Manual segmentation is also subject to inter-observer variability [55,56]; although, some studies have tried to address this issue through multi-reader segmentation. As software to achieve reliable automated segmentation improves and becomes more available, large imaging sets can not only managed efficiently, but segmentation of tumor for radiomic analysis can be performed prospectively as part of the diagnostic radiologist’s clinical workflow [5].

Apart from image acquisition differences, other aspects of heterogeneity within radiomic studies can be seen, accounting for discrepancies in results. For instance, studies investigating the same question (i.e., whether radiomic features can predict the presence of BAP1 mutations) use different phases of CT (i.e., nephrographic vs. excretory vs. unenhanced). Radiomic studies have been inconsistent in the CT phase most predictive of outcomes. As was illustrated above, features derived from CT nephrographic phase was most predictive of BAP-1 mutation status [14]; however, Nguyen et al. found that features from the corticomedullary phase was most predictive of renal mass characterization (e.g., RCC vs. benign) [57]. Just as is performed by the practicing radiologist, the optimal strategy may be to incorporate features from all CT phases into radiomic analysis.

Studies also differ in the extent of feature extraction, with some not obtaining higher order features from image filtration. Additionally, there is variability in the manner through which feature selection is performed, with some but not others employing machine learning to eliminate redundant and/or inconsistent features. Another important, yet underutilized, consideration for feature selection is that predictive model performance may be improved if features related to slice thickness and tumor size are also eliminated [58]. The former is an important consideration with studies relying on multi-institutional databases such as TCIA. With regard to the latter, as radiomics is meant to augment current diagnostic capability, development of radiomic signatures should only involve features that are not easily calculable in the clinical setting.

Thus, for radiomic studies to be reliably compared against each other, standardization of image processing (including acquisition and segmentation); feature extraction; and feature selection needs to be established. Perhaps an international consensus conference can be conducted for this purpose, with stakeholders from different fields outlining guidelines (i.e., radiologists; computer scientists; technicians; physicists; and treating clinicians). Standardization will also ensure that multi-disciplinary collaboration can be robustly performed from high quality and well curated images. Large sample sizes are necessary to improve generalizability of machine learning classifiers. With low sample size (i.e., <1:10 ratio of features: number of patients/tumors in a particular group [5]), overfitting of data can occur, preventing the model from performing well on other types of data, both within and outside a given institution. Additionally, in order to further promote replication of results in other institutions, source code of decision classifiers should be made public, which is not routine practice at present [59].

Currently, the vast majority of current radiomic and radiogenomic studies focus on CT. This approach is sensible at present, given that this imaging modality is the predominant means of evaluating renal masses worldwide. However, with its lack of radiation, MRI has grown in popularity, particularly as more serial imaging is incorporated into kidney tumor evaluation (i.e., active surveillance or treatment response in metastatic disease). The main advantage of MRI is the additional information that can be obtained from a variety of imaging sequences, such as T2 or DWI, which may improve image prediction models by providing additional radiomic features. Only one study reviewed here utilized MRI for computational image feature extraction; it is hoped that additional studies utilizing MRI for radiogenomic analysis will be conducted as experience and/or availability of this imaging modality grows.

In terms of scope of study, radiogenomic analysis thus far has largely focused on molecular features of the tumor itself. However, the tumor exists within a microenvironment that modulates its growth and development. For instance, Zhong et al. [60] identified two subtypes of ccRCC from analysis of the TCGA that differed based on checkpoint inhibitor and lymphocyte expression. These differences in immune-related tumor microenvironment have prognostic relevance; for instance, the subtype with elevated checkpoint inhibitor expression was predicted to have reduced response to immunotherapy. Some preliminary radiogenomic work characterizing the tumor ecosystem has been employed, such as Greco et al. [11] characterizing visceral fat content with ccRCC mutation as well as Marigliano et al. [17] and Lee et al. [23] also incorporating the surrounding normal parenchyma in feature extraction. It is hoped that as the field of radiogenomics evolves along with our understanding of the biology of the tumor microenvironment, additional radiomic analysis of the parenchyma and perinephric fat surrounding a tumor can be performed to establish more comprehensive surrogate imaging biomarkers.

While a clear advantage of establishing imaging biomarkers of underlying genetic activity is that images provide wider anatomical coverage than can be procured by a biopsy sample, many radiogenomic studies still correlate image features of an entire tumor with genetic information from a biopsy specimen. Furthermore, most of the time, the exact location of the biopsy is not known, preventing radiomic analysis of the corresponding area of a tumor to achieve a more optimal association study given genetic intra-tumor heterogeneity [61]. For this reason, the study by Yin et al. [22] was unique in that radiomic analysis was performed at different areas of a single tumor, with each area having distinct genetic testing and thus a known gene expression pattern. Future studies should also perform radiogenomic analysis within tumors as opposed to simply between different tumors. In the era of digital pathology utilizing quantitative image analysis and machine learning, models characterizing spatial heterogeneity of genetic mutations and surrounding microenvironment (i.e., T lymphocyte expression) within a tumor have been developed [62]. Provided that these models can be validated across institutions, they can be integrated into radiomic studies to provide more robust imaging–pathology associations.

Although the majority of presented studies here utilize tumors of different stages in image analysis, the genetic information is generally derived from the primary kidney tumor. That being said, the assumption of genetic homogeneity between the primary tumor and metastatic deposits may not necessarily hold. In a recent study using ClearCode34 to classify primary and metastatic tumor sites into different molecular subtypes (i.e., clear cell type A and B), there was a 43% discordance in subtype between the primary tumor and metastatic deposits within the same patient [63]. On the other hand, for a given patient, the molecular subtypes were similar among different metastatic sites. Thus, future radiogenomic studies incorporating patients with metastatic disease should have tumor sampling from metastatic sites to obtain a more reliable genotype within which to develop image biomarkers for prognostically relevant outcomes such as treatment response. It is clear that feature extraction from radiomic analysis provides more information about a tumor than can be ascertained by any radiologist (i.e., higher order features). However, with greater complexity comes greater abstraction of data from traditional biological or clinical understanding. Seeking to understand higher order features in clinical terms is challenging. However, “de-mystifying” these features can be accomplished through studying associations between qualitative and quantitative image variables. For instance, ill-defined tumor margin is associated with unfavorable genotypes, such as BAP1 mutation; methylation of RUNX3; and SPC gene signature. Determining which radiomic higher order features relate to these qualitative variables will allow for better integration of the literature and to improve clinical relevance of these features.

Given that tumor genetic testing does not often encompass the entire tumor (i.e., biopsy), radiomic analysis may provide additional prognostic information beyond the procured molecular signature [64]. Thus, rather than determining radiomic–genomic correlations alone, studies should incorporate both radiomic and genomic factors into prognostic models. Additional integration of existing clinical predictors and other -omic analysis into these models will also help improve prediction of clinically relevant outcomes. For instance, Zeng et al. [27] demonstrated that a combined radiomic, genomic, transcriptomic, and proteomic model had higher AUC than any single model alone in predicting overall survival of patients with ccRCC. Additionally, Yin et al. [22] showed that a model combining radiomic and clinical features (tumor size; stage; and grade) outperformed a radiomics only model in predicting ccRCC molecular subtype (91.3% vs. 86.96% accuracy). Finally, Huang et al. [26] developed an integrative nomogram of ccRCC survival incorporating tumor stage, gender, and a risk score incorporating both prognostic radiomic and genetic factors.

## 6. Conclusions

Radiogenomics represents the next paradigm shift in diagnostic medicine, and just as with the Human Genome Project, kidney cancer is one of the lead malignancies with which to apply advances from this field. Initial work in radiogenomics of clear cell kidney cancer has been promising, with relationships seen between imaging features and single and multiple gene expression patterns. Not only can image phenotypes be linked to prognostically relevant molecular signatures, but they can also be used to facilitate identification of associated gene expression pathways (i.e., biological basis of image differences) and can augment existing clinico-pathologic nomograms. Establishing non-invasive surrogate imaging biomarkers will no doubt increase the non-invasive diagnostic armamentarium of the clinician, with both prognostic and therapeutic implications, and has been greatly facilitated with radiomics and machine learning, which can elucidate the complex patterns within an image in an objective, quantifiable manner, unlike qualitative scoring by radiologists.

Future directions include feature extraction of the surrounding tumor environment; utilization of modalities other than CT; incorporating spatial tumor genetic heterogeneity in radiomic analysis; and integration of multi-omic (i.e., transcriptomic) and clinical information to create more powerful decision tools. Most importantly, consensus guidelines on radiomic and machine learning analysis need to be employed to facilitate comparison among studies and collaboration among institutions to allow advances in radiogenomics to be implemented in the clinical setting.

## Figures and Tables

**Table 1:** Summary of 20 reviewed articles on radiogenomics in clear cell renal cell carcinoma. Nature of feature extraction is indicated by “Radiologist” if features are scored by one or more radiologists. Elsewise, software derived features are indicated by “Computational”. Number of selected features indicated in parenthesis. TAT (total adipose tissue), VAT (visceral adipose tissue), AUC (area under the curve), OR (odds ratio), HR (hazard ratio), CSS (cancer specific survival), OS (overall survival), PFS (progression free survival).

Author	Title	Year of Publication	Patient #	Feature Extraction (Number)	±Machine Learning	Image Phase Used	Genes Studied	Outcome
Karlo et al. [9]	Radiogenomics of Clear Cell Renal Cell Carcinoma: Associations between CT Imaging Features and Mutations	2014	233	Radiologist (10)	−	CT	BAP1 VHL KD5MC	BAP1 and KD5MC: renal vein invasion (OR 3.50 and 3.89) VHL: ill-defined margin (OR 0.49), nodular enhancement (OR 2.33), intratumoral vasculature (OR 0.51)
Shinagare et al. [10]	Radiogenomics of clear cell renal cell carcinoma: Preliminary findings of the cancer genome atlas–renal cell carcinoma (TCGA–RCC) imaging research group	2015	103	Radiologist (6)	−	Contrast-enhanced CT	BAP1 MUC-4	BAP1: Ill-defined margin and calcification MUC4: Exophytic growth
Greco et al. [11]	Relationship between visceral adipose tissue and genetic mutations (VHL and KDM5C) in clear cell renal cell carcinoma	2021	97	Computational (3)	−	CT	KDM5C vs. VHL	KDM5C higher TAT and VAT area than VHL
Feng et al. [12]	Identifying BAP1 Mutations in Clear-Cell Renal Cell Carcinoma by CT Radiomics: Preliminary Findings	2020	54	Computational (58)	+ (Random Forest)	CT	BAP1	AUC 0.77
Kocak et al. [13]	Machine learning-based unenhanced CT texture analysis for predicting BAP1 mutation status of clear cell renal cell carcinomas	2020	65	Computational (6)	+ (Random Forest)	CT	BAP1	AUC 0.897
Ghosh et al. [14]	Imaging-genomic pipeline for identifying gene mutations using three-dimensional intra-tumor heterogeneity features	2015	78	Computational (1636)	+ (Random Forest)	CT nephrographic phase	BAP1	AUC 0.71
Kocak et al. [15]	Radiogenomics in Clear Cell Renal Cell Carcinoma: Machine Learning-Based High-Dimensional Quantitative CT Texture Analysis in Predicting PBRM1 Mutation Status	2019	45	Computational (10)	+ (Random Forest)	CT	PBRM1	AUC 0.987
Chen et al. [16]	Reliable gene mutation prediction in clear cell renal cell carcinoma through multi-classifier multi-objective radiogenomics model	2018	57	Computational (43)	+ (6 classifier composite)	CT	VHL PBRM1 BAP1	AUC 0.88 0.86 0.93 Mutation status prediction
Marigliano et al. [17]	Radiogenomics in clear cell renal cell carcinoma: correlations between advanced CT imaging (texture analysis) and microRNAs expression	2019	20	Computational (6)	−	CT	miR-21-5p	R^2^ = 0.25 between entropy and change in miR-21-5p expression between tumor and surrounding parenchyma
Cen et al. [18]	Renal cell carcinoma: predicting RUNX3 methylation level and its consequences on survival with CT features	2019	106	Radiologist (9)	−	CT	RUNX3 methylation	High methylation: left side (OR 2.70), ill-defined margin (OR 2.69), intratumoral vascularity (OR 3.29)—AUC of 0.73
Yu et al. [19]	Renal Cell Carcinoma: Predicting DNA Methylation Subtyping and Its Consequences on Overall Survival With Computed Tomography Imaging Characteristics	2020	212	Radiologist (12)	−	CT	Tumor methylation (M1-M3 subtype)	M1: >7 cm (OR 2.45), necrosis (OR 4.76) M2: necrosis (OR 0.047), enhancement (OR 0.083) M3: Long axis > median (OR 0.30), necrosis (OR 3.26)
Jamshidi et al. [20]	The radiogenomic risk score: construction of a prognostic quantitative, noninvasive image-based molecular assay for renal cell carcinoma	2015	70	Radiologist (4)	−	Contrast CT	SPC gene signature	RRS correlation with SPC (R = 0.45), HR 3.32 for CSS after surgery
Jamshidi et al. [4]	The radiogenomic risk score stratifies outcomes in a renal cell cancer phase 2 clinical trial	2016	41	Radiologist (4)	−	Contrast CT	SPC gene signature	PFS: 6 mo (high RRS) vs. >25 mo (low RRS)—After bevacizumab tx OS: 25 mo (high RRS) vs. >37 months (low RRS)
Bowen et al. [21]	Radiogenomics of clear cell renal cell carcinoma: associations between mRNA-based subtyping and CT imaging features	2019	177	Computational (8)	−	CT	mRNA subtyping (m1-m4)	M1: OR 2.1—well-defined margin M3: OR 0.42 (well-defined margin), OR 2.12 (renal vein involvement)
Yin et al. [22]	Integrative radiomics expression predicts molecular subtypes of primary clear cell renal cell carcinoma	2018	8	Computational (4)	+ (Fisher’s linear discriminant analysis)	PET and MRI	Molecular subtype of ccRCC (ccA vs. ccB)	Accuracy of classification—86.96%
Lee et al. [23]	Integrative radiogenomics approach for risk assessment of post-operative metastasis in pathological T1 renal cell carcinoma: a pilot retrospective cohort study	2020	58	Computational (4)	+ (Random Forest)	Contrast CT	Multiple gene-mediated pathways	AUC 0.955—Metastasis
Zhao et al. [24]	Validation of CT radiomics for prediction of distant metastasis after surgical resection in patients with clear cell renal cell carcinoma: exploring the underlying signaling pathways	2021	547	Computational (9)	+ (Logistic regression)	CT	19 gene pathway signatures	AUC 0.84—Metastasis
Lin et al. [25]	Radiomic profiling of clear cell renal cell carcinoma reveals subtypes with distinct prognoses and molecular pathways	2021	160	Computational (122)	+ (Consensus clustering)	Unenhanced CT	VHL, MUC16, FBN2, and FLG Cell cycle related pathways	C1: Lower OS and PFS than C2 and C3 C1: Lower VHL expression C3: Higher FBN2 expression
Huang et al. [26]	Exploration of an integrated prognostic model of radiogenomics features with underlying gene expression patterns in clear cell renal cell carcinoma	2021	205	Computational (4)	+ (LASSO/SVM for feature selection, random forest for classification)	Contrast CT	Gene modules	AUC 0.837, 0.806 and 0.751—1-, 3-, and 5-year OS (combined radiogenomic model)
Zeng et al. [27]	Integrative radiogenomics analysis for predicting molecular features and survival in clear cell renal cell carcinoma	2021	207	Computational (4)	+ (Random Forest)	Contrast CT	VHL, BAP1, PBRM1, SETD2, molecular subtypes (m1–m4)	AUC 0.846—5-year OS (Combined radiogenomic model)

The # refers to number (as in number of patients).
